# The role of surgery in the treatment of neuroblastoma metastases at rare sites

**DOI:** 10.1007/s00432-023-05147-6

**Published:** 2023-07-19

**Authors:** Simon Scherer, Benjamin F. B. Mayer, Markus Dietzel, Michael Esser, Steven W. Warmann, Peter Lang, Martin U. Schuhmann, Andreas Schmidt, Jörg Fuchs

**Affiliations:** 1 Department of Pediatric Surgery and Pediatric Urology, University Children’s Hospital Tuebingen, Hoppe-Seyler-Str. 3, 72076 Tübingen, Germany; 2 Department of Diagnostic and Interventional Radiology, University Hospital Tuebingen, Hoppe-Seyler-Str. 3, 72076 Tübingen, Germany; 3 Department of General Pediatrics, Hematology and Oncology, University Children’s Hospital Tuebingen, Hoppe-Seyler-Str. 1, 72076 Tübingen, Germany; 4 Department of Neurosurgery, University Hospital Tuebingen, Hoppe-Seyler-Str. 3, 72076 Tübingen, Germany

**Keywords:** Neuroblastoma metastases, Surgical therapy, Penis metastasis, Pancreas, Bone, Proximal ulna, Pediatric metastatic disease

## Abstract

**Purpose:**

Treatment of neuroblastoma metastases usually consists of chemotherapy and irradiation. However, in selected cases, surgical treatment is also indicated. In this study, we present three cases of patients with neuroblastoma metastases at rare sites that underwent surgery.

**Materials and methods:**

We retrospectively analyzed data of patients who underwent surgery for neuroblastoma at our department of Pediatric Surgery and Pediatric Urology at the University Children’s Hospital in Tuebingen and selected those patients who had surgery explicitly for a metastasis.

**Results:**

Between 2002 and 2020, 277 children underwent surgical treatment for neuroblastoma. Three cases with metastases at exceptional sites are presented here after therapy according to protocols. One patient had a penile metastasis and received surgery including a plastic reconstruction. The patient showed no signs of erectile or urinary dysfunction at follow-up. Another patient had a metastasis in the proximal ulna, which remained vital even after exhausted treatment after two relapses. Afterward there was no restriction of movement of the extremity. The third patient had, amongst others, metastases to the pancreatic body and to the liver. Both were surgically removed during primary tumor resection. This patient died after local tumor relapse. The other two patients showed no evidence of tumor relapse after a follow-up of 18 and 17 months, respectively.

**Conclusion:**

Although children with neuroblastoma often present with metastases, there is no recommendation for surgical treatment other than diagnostic biopsies. In case of persistence of metastasis or after exhaustion of high-risk therapy, surgical resection must be considered.

## Introduction

Neuroblastoma is the most common extracranial solid tumor in children (Pinto et al. 2015). About half of the children with neuroblastoma present with metastases at the time of diagnosis and therefore are classified as stage 4 (surgically-based INSS) or M (radiology-based INRGSS) (DuBois et al. 1999; Simon 2019). Event-free survival rates in metastatic disease are poor, ranging from 15 to 33% (Hayes et al. 1983; DuBois et al. 1999; Kammen et al. 2001). DuBois et al. described in 648 patients with stage 4S and 4 neuroblastoma the following most common locations for hematogenous metastases with a somewhat variable incidence depending on the literature: bone marrow (70.5 to 86%), bone (55.7 to 66%), lymph nodes (19 to 30.9%), liver (17 to 29.6%) and intracranial/orbit (18.2%) (DuBois et al. 1999; Sohara et al. 2005; Simon 2019). Multimodal therapy of the primary tumor is performed according to established study protocols in which surgery is an integral part and an essential factor in the prognosis of the affected children. Regarding the therapy of neuroblastoma metastases, there is no consensus among the different protocols. Furthermore, especially in highly aggressive metastases, the therapeutic approach is not clearly defined, and the role of surgical treatment remains unclear (Berthold 2004, Simon 2019). In this study, we present three cases of rare sites of metastases of neuroblastoma and their surgical therapies: one case with penile metastasis, which are generally rare in all solid tumor entities in children; one case with a metastasis in the proximal ulna refractory to therapy, and one case with multiple metastases especially of the pancreas tail and liver.

## Materials and methods

### Patients and ethical considerations

The department of Pediatric Surgery and Pediatric Urology at the University Children’s Hospital in Tuebingen, Germany, is a national surgical reference center for different extracranial solid tumor entities in children, including neuroblastoma. All patients in this study were operated on by the senior author (JF). We retrospectively analyzed all patients who underwent surgery for neuroblastoma at our department between March 2002 and October 2022. Staging was performed in accordance with INRS and INSS criteria (Shimada et al. 1999; Monclair et al. 2009). Risk stratification was based on the national guideline by the German Society for Pediatric Oncology and Hematology (Simon 2019). The decision for surgical treatment of neuroblastoma metastases was made on an interdisciplinary basis in the German national MDT, and individual circumstances were considered. Written informed consent for surgical treatment was obtained from the patient’s parents. Patient data, therapy, indication for surgery, intraoperative and pathology findings, and outcome data were analyzed retrospectively. Data were acquired and processed according to the latest version of the “World Medical Association Declaration of Helsinki–Ethical Principles for Medical Research Involving Human Subjects”. The study was approved by the local ethical committee (project number 013/2022BO2).

## Results

Between March 2002 and October 2022, surgical treatment of neuroblastoma was performed in 277 patients. The metastases of neuroblastoma were treated surgically in three patients. Patient characteristics are summarized in Table 1.

**Table 1:** Patient demographics and tumor profiles

Patient no	Sex	Age at initial neuroblastoma diagnosis	INSS stage at initial presentation	INRGSS stage	MYCN amplification	1p deletion	Primary tumor site	Macroscopic complete resection	Pathological findings of resected metastasis	Classification
1	m	6 mo	4	M	No	No	Left adrenal gland	Yes	Penile metastasis: 10 g heavy nodular soft tissue excidate, 4.2 × 2.2 × 1.9 cm manifestation of differentiating neuroblastoma. No evidence of necrosis. Low mitotic and karyorrhexis index according to INPC classification	Hughes: Neuroblastoma 1a. Regression grade 4. Differentiation grade 2
2	m	2 y 6 mo	4	M	No	No	Left adrenal gland	Yes	Ulnar bone metastasis: 8.6 × 3.1 × 1.6 cm soft tissue excidate. Soft tissue manifestation of neuroblastoma with approximately 90% tumor viability	Hughes: Neuroblastoma grade 1a. Regression grade 4. Differentiation grade 2
3	w	6 y 2 mo	4	M	Yes	No	Right adrenal gland	Yes	Pancreatic metastasis: 1.1 cm diameter manifestation of the Lineuroblastoma, removed in sano, approximately 50% vital. The margins are tumor-free. Poorly differentiated, low stroma neuroblastoma with low mitosis and karyorrhexis index according to INPC with severe pleomorphism, partly in the form of pancreatic, liver and lymph node metastases, with hemangio- and lymphangioinfiltration. Liver metastasis: 80% vital, predominantly increased mature imposing compared to primary tumor	Hughes: Neuroblastoma grade 3. Regression grade 4. Differentiation grade 4

*INSS* International Neuroblastoma Staging System, *INRGSS* International Neuroblastoma Risk Group Staging System

### Case 1

**Fig. 1:**
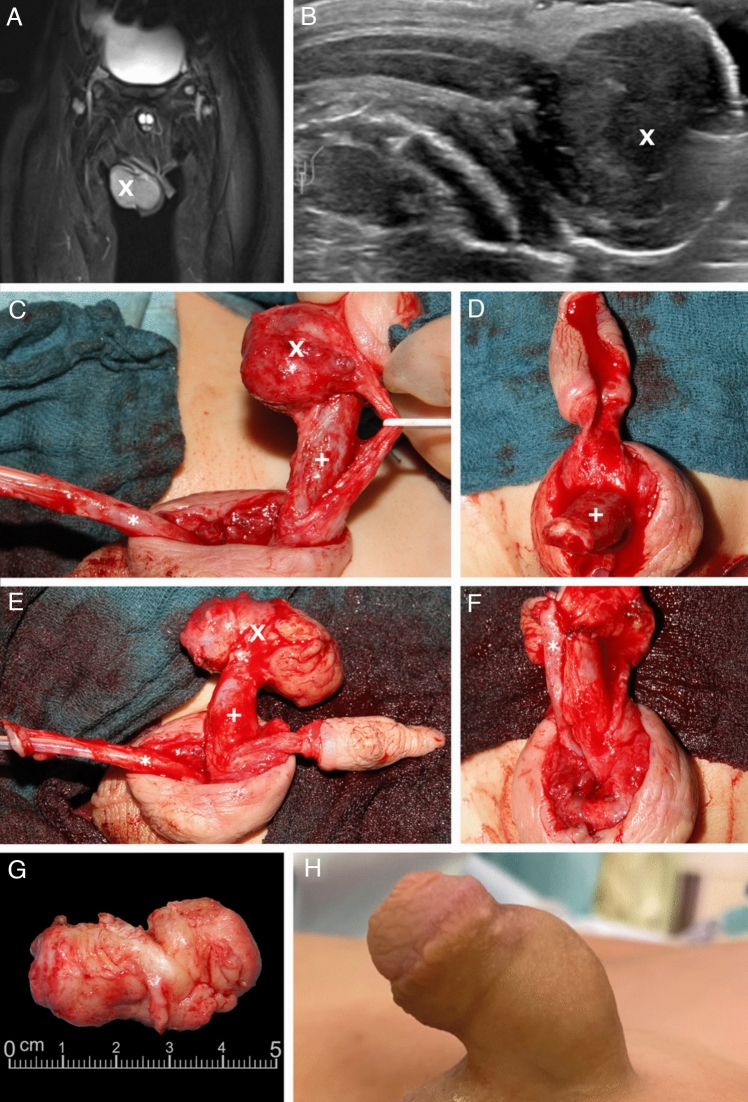
A 26-month-old boy with neuroblastoma stadium 4 and a metastasis to the penile shaft. MRI post-contrast with fat saturation, coronary (**A**). Sonographic longitudinal view demonstrating the relation of the metastasis to the corpus cavernosum (**B**). Surgical site (**C**, **D**, **E**, **F**). The resected metastasis (**G**). Postoperative outcome 18 months after metastatic surgery (**H**). X = Penile metastasis. +  = Corpora cavernosa. * = Urethra.

A 26-month-old boy with neuroblastoma stage IV (intermediate-risk group, negative MYCN amplification, negative 1p deletion) was referred to our department. MIBG scintigraphy showed a mIBG-positive primary tumor in the left adrenal region, a skin metastasis on the right-frontal skull and a penile metastasis adjacent to the glans with a size of 4.2 cm/1.7 inch (Fig. 1) and infiltration of the corpus spongiosum and the urethra. After initial diagnosis, he was treated with 2 cycles of N4 chemotherapy according to the NB2016 registry protocol (Berthold 2004). After chemotherapy, the primary tumor was no longer detectable. The patient was referred to the author’s center for surgical management of the growing penile metastasis. Due to the high probability of side effects such as erectile dysfunction and scarring of the penile tissue after local irradiation of the penis, the national MDT recommended surgical therapy of the penile neuroblastoma metastasis. As a secondary finding, we saw a condition of coronal hypospadias with a megalourethra.

Surgery was performed under general endotracheal anesthesia. A transurethral balloon catheter was inserted under sterile conditions. The transition region was incised from glans to penile shaft, and a degloving of the penile skin from the shaft was performed. Step by step, the corpus spongiosum was separated from the corpora cavernosa. The corpus spongiosum was found to be infiltrated by the metastasis on a length of 2 cm. The corpus spongiosum and urethra were mobilized, and the intact urethra was pulled up to the tip. Subsequently, the neurovascular bundle was dissected using quadruple magnifying glasses, and finally, the metastasis was resected from the glans with a resection margin of 2–3 mm. Furthermore, the metastasis was removed from the corpus spongiosum so that the penis was divided into 3 essential parts according to the Mitchell technique: the glans with the neurovascular bundle, the corpus spongiosum, and the corpora cavernosa (Mitchell and Bagli 1996). A complete resection of the metastasis could be performed, and the corpora cavernosa were sutured to the glans. Non-absorbable sutures were used for this step, and the urethra was brought to the tip of the glans. Extensive reconstruction of the glans with opposing mattress sutures was performed. Excess penile shaft skin was resected, and the remaining skin reconstructed. After the surgery, the penis was found to be well perfused. Dressing was applied in the same way as after surgical hypospadias repair: a film dressing was applied as the first layer and a gauze bandage was wrapped around the penis shaft as the second layer.

The postoperative course was uneventful. After 10 days, the transurethral catheter was removed, and we saw repeated spontaneous voiding and no clinical irregularities. The patient was discharged the following day with dry wound conditions and in good general condition. At follow-up 18 months after surgery, the patient was alive without signs of tumor relapse, was voiding regularly without problems and had regular erections according to the parents.

### Case 2

**Fig. 2:**
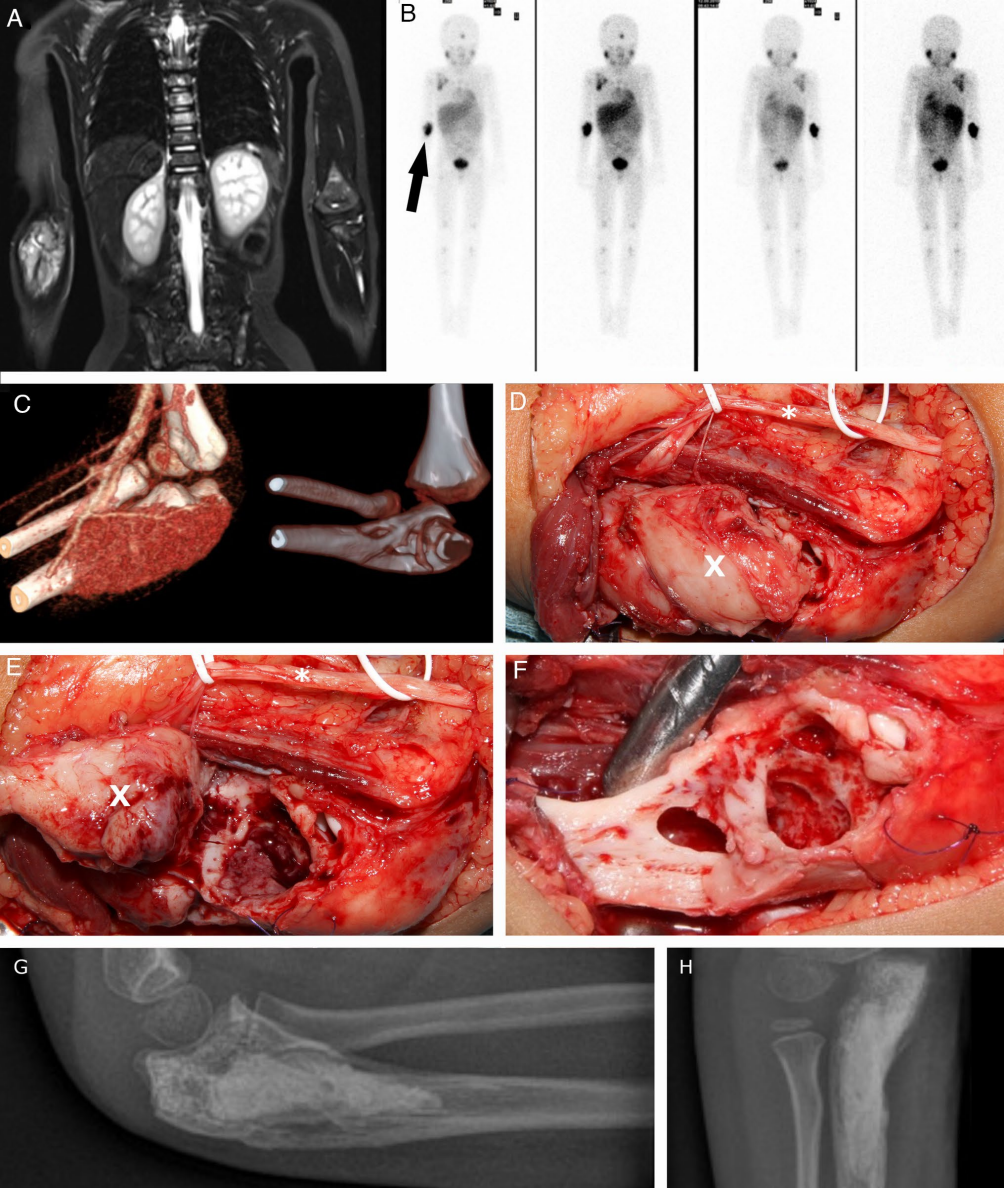
A 6-year-old boy with neuroblastoma stadium and, among other, metastatic disease to the right proximal ulna. Initial diagnosis on whole-body MRI, T2 weighting, TIRM, coronary (**A**). Tumor scintigraphy, 119 MBq, 123 J mIBG. Illustrative marking of the left ulnar metastasis by arrow (**B**). 3-dimensional CT reconstruction of the left elbow joint with metastatic hypervascularization in the proximal ulna (**C**). Perioperative visualization of the left proximal ulna after slinging of the ulnar nerve using white rubber loops; * = Ulnar nerve. X = Metastasis of left ulna (**D**). The left proximal ulna after mobilization of the metastasis and view of the bony cavity; * = Ulnar nerve. X = Metastasis of left ulna (**E**). View of the bony cavity after complete removal of the metastasis; suture marking (blue) of the muscle attachments of the superficial flexors (**F**). Postoperative conventional X-ray control in two planes after complete removal of the bone metastasis and filling of the bony cavity with bone substitute material (**G**, **H**)

A 6-year-old boy with evidence of a second relapse of a neuroblastoma stadium 4 (high-risk group, negative MYCN amplification, negative 1p deletion; initial diagnosis: October 2016, first relapse: August 2017, second relapse: March 2019) was referred to the department of Pediatric Oncology and Hematology for stem cell transplantation. At diagnosis, the patient had a mIBG-positive tumor manifestation originating from the left adrenal gland with multiple mIBG-positive bone metastases and a suspicious lymph node in the right axilla with a diameter of 0.14 cm/0.55 inch (Fig. 2). The primary treatment consisted of chemotherapy according to Children’s Oncology Group protocol, tumor resection, autologous bone marrow transplantation, and radiotherapy (Pinto et al. 2015, Irwin et al. 2021). A relapse of the tumor was detected during routine follow-up examination, and mIBG therapy with 2 additional cycles of chemotherapy was administered. A few weeks later, a second relapse was detected. The following treatment included 1 cycle of chemotherapy with ifosfamide, carboplatin and etoposide, a second regimen of mIBG therapy, haploidentical stem cell transplantation, local radiation (the right elbow and right axilla were irradiated with a dose of 40 Gy in 20 fractions and the right proximal femur, as well as the cranial bone were irradiated with a dose of 36 Gy in 18 fractions), and immunotherapy with dinutuximab. After therapy of the second relapse, the metastasis of the right ulna and the lymph node in the right axilla were still visible on imaging and appeared to be vital. The patient’s main objectives were discussed in the local MDT, and it was decided to resect both metastases as part of the multimodal therapy.

Under general endotracheal anesthesia, a tourniquet was applied around the right upper arm. The surgical removal was performed together with a pediatric neurosurgeon. An opposing l-shaped incision was made in the area of the ulnar metastasis, which was followed by nerve visualization and preparation of the muscle insertions. After transection of the muscle attachment at the ulnar process, the metastasis was visible and could then be separated from the radial and ulnar arteries. A gross total resection with removal of approximately 98% of the tumor mass was performed using a sharp spoon and raspatory instrument. The bone structures proximal and distal to the metastasis were visualized, and the integrity of the elbow joint was assessed. It was decided to use bone cement and osteostimulative bone substitute with silicate substitution (Actifuse^®^, Baxter Inc., Deerfield, IL/USA) to fill the bone defect. In addition, a fibrin sealant patch (TachoSil^®^, Takeda Pharmaceutical Company Limited, Tokyo/Japan) was applied. Then, the muscle attachments were reconstructed, the ulnar nerve repositioned, and the wound closed layer by layer with insertion of a drain. After resection of the ulnar metastasis, the axillary lymph node metastasis was resected. At the end of the surgery, an upper arm cast was attached, and the patient was transferred to the pediatric surgical ward. Before discharge, the cast was changed to an elbow orthosis (Epico ROM^®^, Medi GmbH & Co. KG, Bayreuth/Germany), with which immobilization of the elbow joint at an angle of 100° was established. At follow-up, the freedom of mobility of the left elbow joint could rapidly be improved until after six weeks the range of motion was only limited at maximum flexion and supination. At this time, imaging was performed using mIBG scintigraphy. No more mIBG-positive tumor sites or metastases could be detected. At follow-up 2.5 years after surgical treatment of the metastases, no signs of tumor relapse were noted, and according to the family, the patient is doing well.

### Case 3

**Fig. 3:**
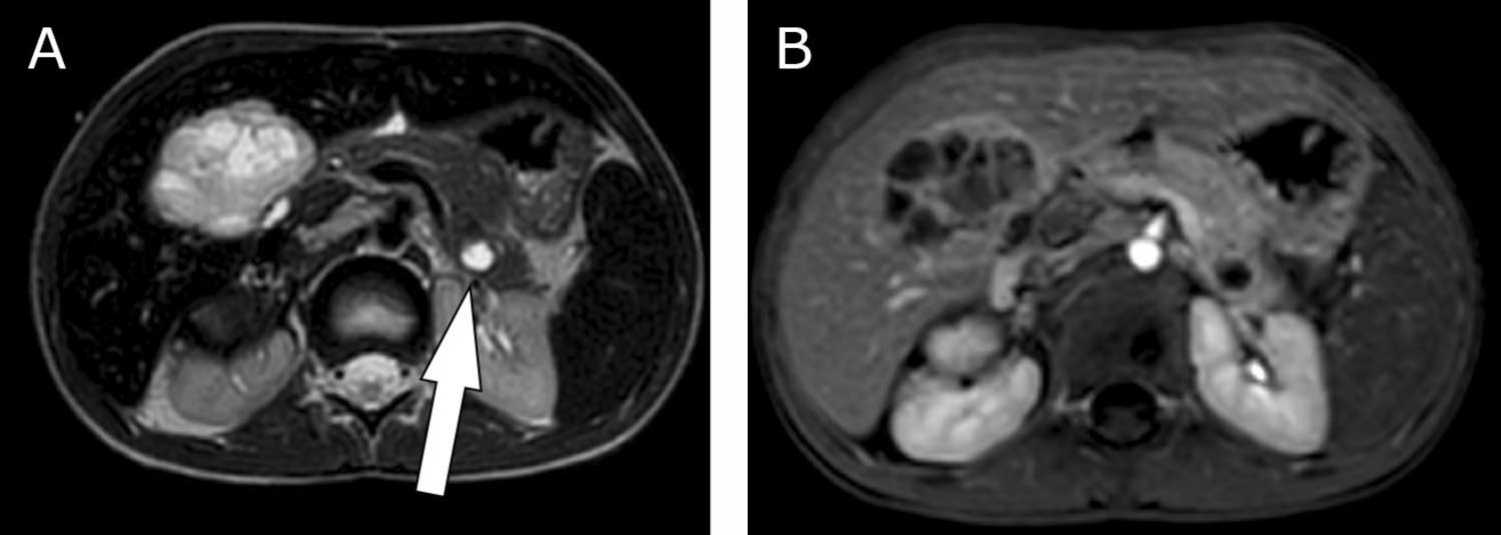
A 6-year-old girl with neuroblastoma stadium 4 and metastasis to the pancreatic tail and liver. MRI (T2) of the abdomen, transversal plane: hyperintense focus of the pancreatic tail and visible liver metastasis in segment 4 (**A**), Illustrative marking of the left pancreatic metastasis by arrow. MRI of the abdomen, Contrast dynamics, early phase, marginal low contrast uptake (**B**).

A 6-year-old girl was referred to our department with a neuroblastoma stadium 4 (high-risk group, MYCN amplification, negative 1p deletion) originating from the right adrenal gland (and the right sympathetic trunk) with encasement of the large abdominal vessels and infiltration of the diaphragm. The patient had metastatic disease to intra-abdominal and left cervical lymph nodes, the pancreatic tail with a diameter of 1.1 cm/0.43-inch, the liver, to various bones, and in the bone marrow (Fig. 3). After initial diagnosis, the patient was treated with 5 N5/N6 chemotherapy cycles according to the GPOH registry protocol (Berthold 2004). The patient was referred to the author’s department for resection of the primary tumor of the right adrenal gland and the liver and pancreas metastases. The national MDT’s decision to resect the metastases was made because the metastases had shown growth and high viability in the MRI, based on advanced diagnostic imaging value and diffusion weighted imaging as well as image-defined risk factors (Monclair et al. 2009, 2015). In addition, non-surgical therapy had shown no effect. A transverse upper abdominal laparotomy was realized, and a complex gross total resection was performed with release of all encased vessels without organ injuries. The excision of the metastases of the pancreatic tail and the liver was realized by pancreatic tail resection and right hemihepatectomy without any complications. Intraoperatively, the remaining organ mass of the liver was estimated to be 55%, and the pancreas still had approximately 60% of its original volume. During the postoperative course, there was no evidence of pancreatic or bile leakage as well as no clinical or laboratory signs of endocrine or exocrine pancreatic insufficiency. The patient died 6.5 months after surgery due to local tumor relapse and rapid tumor progression under palliative treatment in a hospital near her home.

## Discussion

In this study, the surgical treatment of 3 patients with metastatic diseases at rare sites in neuroblastoma, the individual therapies, and the conclusions drawn from the authors' experience is presented. Although the majority of children with neuroblastoma present with metastatic disease at the time of diagnosis, current protocols indicate surgical treatment of metastases only for diagnostic confirmation by biopsy. The standard treatment of choice for metastases of neuroblastoma consists of chemotherapy and radiotherapy. The staging and the presence of minimal residual disease after primary tumor resection ultimately determine the exact procedure.

As in the cases presented here, resection of metastases is always part of a personalized medical concept that should be established in the context of national or international MDT. In general, metastases in neuroblastoma decrease under non-surgical therapy. In 2 of this study’s patients, the standard therapy, chemotherapy and radiotherapy had been exhausted, the metastases still appeared vital on imaging or an extraordinarily large tumor mass of manifested itself as a liver metastasis. In the third patient, the national MDT decided against standard therapy and in favor of surgical therapy of the metastasis because of the unusual location on the penile shaft, weighing the risks of therapy according to standard protocol the lifelong side effects of radiation therapy. In summary, in these very rare and particular cases, non-surgical therapy was unsuccessful, so that the exceptional indication for metastatic surgery was made after individual consideration. All surgical concepts for these patients were reconstructive and not mutilating.

There are typical sites for metastases in neuroblastoma, but the variability of their location is described in a study reporting over 500 metastatic sites found at diagnosis in 43 high-risk neuroblastoma patients (Polishchuk et al. 2014). The most common metastases are found in the bone marrow, but their presence does not seem to have prognostic value (Wang et al. 2019). The biologic mechanisms involving invasion of surrounding tissue, penetration of blood vessels and arrest in the bone marrow cavity have been described in detail (Sohara et al. 2005; Modak and Cheung 2010; He et al. 2021). Bone metastases are supposed to have an unfavorable prognostic value and are the second most common site of metastatic disease in neuroblastoma. Another study described a mortality rate for patients with bone metastasis of more than 93% (Golen et al. 2006). There is a single case report of a neuroblastoma originating from the pancreas (Kumar et al. 2010), as well as 9 reported pancreatic metastases with 1 that occurred in combination with an ovarian metastasis (Kim et al. 2008; Rosenbaum et al. 2013; Sakai et al. 2021). As described also, the pancreas is an unusual site of metastasis of neuroblastoma even though other solid extracranial tumors in children, such as osteosarcoma and rhabdomyosarcoma, do metastasize to the pancreas (Rosenbaum et al. 2013), and in contrast to liver metastases, which are among the most common localization of metastatic neuroblastoma (DuBois et al. 1999). Metastases to the penis are generally very rare across all tumor entities, and to our knowledge, this is the first report of a neuroblastoma metastasizing to the penis and generally of a penile metastasis of an extracranial solid tumor in children (Mearini et al. 2012).

In the 2017 GPOH guidelines, irradiation of metastatic lesions is currently justified in the case of singular or few residual metastases persisting after treatment according to protocol, after individual consideration. There is no defined differentiation with regard to localization or size (Simon et al. 2017; Simon 2019). However, the location and time of occurrence of metastases appear to be relevant since improvements in overall survival may increase the chance and probability that neuroblastoma metastases can present at unusual sites. This metastatic spread could be due to highly aggressive subclonal population of tumor of cells, which could indicate that these tumors are likely to be associated with prognostically unfavorable outcomes (Pandian et al. 2015). The COG protocol for high-risk neuroblastoma recommends fractionated radiotherapy given in 12 equal fractions of 1.8 Gy to the primary site and to all mIBG avid metastatic sites after consolidation chemotherapy (Kreissman et al. 2013). Accordingly, the 21 Gy have been recommended for several years and have also been validated (Casey et al. 2016). However, optimized doses are currently being sought due to side effects of radiation (Casey et al. 2019). Yet, with gross disease high-risk neuroblastoma, the American National Cancer Institute with its PDQ^®^ also based on Casey et al. recommends a dose of 30 to 36 Gy (Casey et al. 2018). The American protocol addresses, that in cases where diffuse bone metastases remain after induction chemotherapy, high-dose chemotherapy followed by re-evaluation is performed before consolidative radiotherapy. Currently, no official recommendation has yet been made in the German protocol.

Even though the guidelines are based on current evidence and are continuously adapted, guidelines exist only for radiotherapy of neuroblastoma metastases and not for their surgical treatment. The role of surgical treatment of metastases in neuroblastoma remains unclear in all established treatment protocols. This is with exception of metastases to the central nervous system, where neurosurgical intervention is recommended to decrease edema, control hemorrhage, and remove bulky tumor before radiotherapy. We can assume that surgical treatment of metastases, if performed in specialized centers, can be performed safely and may represent another therapeutic option in the treatment of neuroblastoma, as described for lung metastases previously, and may have a positive impact on individual prognosis (Fuchs et al. 2012).

## Conclusion

Although children with neuroblastoma often present with metastases, there are no clear recommendations regarding surgical treatment of metastases that persist, increase, or remain vital after standard therapy except for diagnostic confirmation by biopsy. Often, the metastases are irradiated with potential side effects. However, surgical treatment can be indicated in selected cases and especially for metastases at exceptional sites, where there is a contraindication due to radiation damage. In the author’s opinion, metastatic surgery is also justified and is the therapy of choice if the tumor is still vital and has been treated and irradiated according to the guidelines, and where the risk of mutilation is low. Two children in this series with negative MYCN, no 1p deletion and after GTR survived, suggesting that surgical treatment of metastases might be favorable especially in cases of MYCN negative neuroblastoma.

## Data Availability

The datasets generated during or analyzed during the current study are available from the corresponding author on reasonable request.

## References

[CR1] Berthold F (2004) NB2004 Trial protocol for risk adapted treatment of children with neuroblastoma. Gesellschaft für Pädiatrische Onkologie und Hämatologie (GPOH). https://www.gpoh.de/sites/gpoh/kinderkrebsinfo/content/e1676/e9032/e68518/e206421/download7674/SeitenausNB_2004_1.00_klein_ger.pdf. Accessed 06 Jan 2023

[CR2] Casey DL, Kushner BH, Cheung N-KV, Modak S, LaQuaglia MP, Wolden SL (2016). Local control with 21-Gy radiation therapy for high-risk neuroblastoma. Int J Radiat Oncol Biol Phys.

[CR3] Casey DL, Kushner BH, Cheung NV, Modak S, LaQuaglia MP, Wolden SL (2018). Dose-escalation is needed for gross disease in high-risk neuroblastoma. Pediatr Blood Cancer.

[CR4] Casey DL, Kushner BH, Cheung N-KV, Modak S, Basu EM, Roberts SS (2019). Reduced-dose radiation therapy to the primary site is effective for high-risk neuroblastoma: results from a prospective trial. Int J Radiat Oncol Biol Phys.

[CR5] DuBois SG, Kalika Y, Lukens JN, Brodeur GM, Seeger RC, Atkinson JB (1999). Metastatic sites in stage IV and IVS neuroblastoma correlate with age, tumor biology, and survival. J Pediatr Hematol Oncol.

[CR6] Fuchs J, Seitz G, Handgretinger R, Schäfer J, Warmann SW (2012). Surgical treatment of lung metastases in patients with embryonal pediatric solid tumors: an update. Seminars in pediatric surgery.

[CR7] Hayes FA, Green A, Hustu HO, Kumar M (1983). Surgicopathologic staging of neuroblastoma: prognostic significance of regional lymph node metastases. J Pediatr.

[CR8] He B, Mao J, Huang L (2021). Clinical characteristics and survival outcomes in neuroblastoma with bone metastasis based on SEER database analysis. Front Oncol.

[CR9] Irwin MS, Naranjo A, Zhang FF, Cohn SL, London WB, Gastier-Foster JM (2021). Revised neuroblastoma risk classification system: a report from the children’s oncology group. J Clin Oncol.

[CR10] Kammen BF, Matthay KK, Pacharn P, Gerbing R, Brasch RC, Gooding CA (2001). Pulmonary metastases at diagnosis of neuroblastoma in pediatric patients. Am J Roentgenol.

[CR11] Kim EY, Yoo S-Y, Kim JH, Sung KW (2008). Pancreatic metastasis in a child suffering with treated stage 4 neuroblastoma. Korean J Radiol.

[CR12] Kreissman SG, Seeger RC, Matthay KK, London WB, Sposto R, Grupp SA (2013). Purged versus non-purged peripheral blood stem-cell transplantation for high-risk neuroblastoma (COG A3973): a randomised phase 3 trial. Lancet Oncol.

[CR13] Kumar HR, Sandoval JA, Lovell MA, Fenton LZ, Bealer JF (2010). Primary pancreatic neuroblastoma: an unusual tumor in infancy. J Pediatr Surg.

[CR14] Mearini L, Colella R, Zucchi A, Nunzi E, Porrozzi C, Porena M (2012). A review of penile metastasis. Oncol Rev.

[CR15] Mitchell ME, Bagli DJ (1996). Complete penile disassembly for epispadias repair: the mitchell technique. J Urol.

[CR16] Modak S, Cheung N-KV (2010). Neuroblastoma: therapeutic strategies for a clinical enigma. Cancer Treat Rev.

[CR17] Monclair T, Brodeur GM, Ambros PF, Brisse HJ, Cecchetto G, Holmes K (2009). The International Neuroblastoma Risk Group (INRG) staging system: an INRG task force report. J Clin Oncol.

[CR18] Monclair T, Mosseri V, Cecchetto G, De Bernardi B, Michon J, Holmes K (2015). Influence of image-defined risk factors on the outcome of patients with localised neuroblastoma. A report from the LNESG1 study of the European international society of paediatric oncology neuroblastoma group. Pediatr Blood Cancer.

[CR19] Pandian V, Ramraj S, Khan FH, Azim T, Aravindan N (2015). Metastatic neuroblastoma cancer stem cells exhibit flexible plasticity and adaptive stemness signaling. Stem Cell Res Ther.

[CR20] Pinto NR, Applebaum MA, Volchenboum SL, Matthay KK, London WB, Ambros PF (2015). Advances in risk classification and treatment strategies for neuroblastoma. J Clin Oncol.

[CR21] Polishchuk AL, Li R, Hill-Kayser C, Little A, Hawkins RA, Hamilton J (2014). Likelihood of bone recurrence in prior sites of metastasis in patients with high-risk neuroblastoma. Int J Radiat Oncol Biol Phys.

[CR22] Rosenbaum DG, Abramson SJ, DeLappe E, Teruya-Feldstein J, La Quaglia MP, Fox JJ (2013). Pancreatic involvement in neuroblastoma with radiologic-pathologic correlation: a single-institution experience. Am J Roentgenol.

[CR23] Sakai S, Nomura K, Abe T, Hayashi K, Tsutsuno T, Mizushima H (2021). Neuroblastoma with ovarian and pancreatic metastasis. J Pediatr Surg Case Rep.

[CR24] Shimada H, Ambros IM, Dehner LP, Ji H, Joshi VV, Roald B (1999). The international neuroblastoma pathology classification (the Shimada system). Cancer Interdisciplinary Int J Am Cancer Soc.

[CR25] Simon T (2019) S1-Leitlinie 025-008 Neuroblastom. Arbeitsgemeinschaft der Wissenschaftlichen Medizinischen Fachgesellschaften (AWMF). https://www.awmf.org/uploads/tx_szleitlinien/025-008l_S1_Neuroblastom_2019-07_01.pdf. Accessed 06 Jan 2023.

[CR26] Simon T, Hero B, Schulte JH, Deubzer H, Hundsdoerfer P, von Schweinitz D (2017). 2017 GPOH guidelines for diagnosis and treatment of patients with neuroblastic tumors. Klin Padiatr.

[CR27] Sohara Y, Shimada H, DeClerck YA (2005). Mechanisms of bone invasion and metastasis in human neuroblastoma. Cancer Lett.

[CR28] van Golen CM, Schwab TS, Kim B, Soules ME, Su OhS, Fung K (2006). Insulin-like growth factor-I receptor expression regulates neuroblastoma metastasis to bone. Can Res.

[CR29] Wang Z, Sun H, Li K, Yao W, Dong K, Ma Y (2019). Prognostic factor analysis of stage 4S neuroblastoma in infant patients: a single center study. J Pediatr Surg.

